# Time Till Viral Clearance of Severe Acute Respiratory Syndrome Coronavirus 2 Is Similar for Asymptomatic and Non-critically Symptomatic Individuals

**DOI:** 10.3389/fmed.2021.616927

**Published:** 2021-03-26

**Authors:** Nitya Kumar, AbdulKarim AbdulRahman, Salman AlAli, Sameer Otoom, Stephen L. Atkin, Manaf AlQahtani

**Affiliations:** ^1^Royal College of Surgeons in Ireland, Manama, Bahrain; ^2^Bahrain Defense Force Hospital, Manama, Bahrain

**Keywords:** SARS-CoV-2, asypmtomatic, viral clearance, COVID-19, viral convergence

## Abstract

Despite the modeled estimations of the burden of asymptomatic spread, the duration of viral positivity and infectiousness of severe acute respiratory syndrome coronavirus 2 (SARS-CoV-2) remains understudied. The objective of the present study was to estimate and compare the time till viral clearance of SARS-CoV-2 in asymptomatic and non-critical symptomatic individuals. We studied 184 SARS-CoV-2-positive participants, of whom 145 were asymptomatic. Our analysis uncovered that time till viral negativity is similar for subclinical [median time till viral clearance: 11 days, interquartile range (IQR): 8, 14] and overt infections (median: 11 days, IQR: 9, 14) after controlling for age and sex. This has implications in understanding the period of infectivity for SARS-CoV-2 in order to plan adequate public health measures to control the community spread.

## Introduction

Asymptomatic severe acute respiratory syndrome coronavirus 2 (SARS-CoV-2) infections remain highly under-diagnosed (1) and are partly responsible for the recent uptick in coronavirus disease 2019 (COVID-19) cases following the reopening of business, universities, and schools around the globe, especially in the United States (2, 3) and the United Kingdom (4). Initial studies on asymptomatic spread report that it accounts for 6% to 30% of SARS-CoV-2 infections in various settings (5, 6). Although median time till viral clearance in symptomatic individuals has been reported to be between 10 and 14 days (7, 8), time kinetics of viral clearance and the duration of infectiousness in asymptomatic individuals remains poorly understood. The present analysis aimed to estimate time till viral clearance in asymptomatic individuals and see how it compares with that of non-critically symptomatic ones.

## Methods

### Study Population

Study population consisted of individuals who arrived in Bahrain from Iran and Egypt between 25 February 2020 and 14 March 2020 and tested positive for SARS-CoV-2 upon mandatory COVID-19 screening prior to entering the country.

### Screening Procedure and Outcome Assessment

Information on reported COVID-19 symptoms was gathered from the study subjects who underwent nasopharyngeal swab testing for SARS-CoV-2 using reverse transcriptase–polymerase chain reaction (RT-PCR) test. After screening, all of the study subjects served mandatory quarantine in isolation wards (or hospitals, when symptomatic), until viral clearance occurred—defined as two consecutive negative RT-PCR test results 24 h apart. We used Thermo Fisher Scientific (Waltham, MA) TaqPath 1-Step RT-qPCR Master Mix, CG on the Applied Biosystems (Foster City, CA) 7500 Fast Dx RealTime PCR Instrument for conducting the PCR assay. The E gene was used and targeted for the assay, and when detected, the test was confirmed by RdRP and N genes. E gene Ct values > 40 were considered negative. For quality control, both positive and negative controls were used. Time until viral clearance was ascertained by testing the individuals every 2 days till viral clearance occurred. Any individuals who developed symptoms while undergoing quarantine were counted as symptomatic.

### Statistical Analyses

Age, sex, and presence of symptoms in the participants have been reported as proportions. Odds of being symptomatic were estimated using a logistic regression model, which was adjusted for categories of age and sex. Time till viral clearance has been reported as medians and interquartile ranges (IQRs) (computed from Kaplan–Meier analysis), and difference across categories of covariates was tested using a log rank test. Hazards of viral clearance were estimated using a Cox proportional hazards model.

## Results

The total number of participants in the study was 184, which included 132 subjects who tested positive at screening and 52 out of 2,526 people who tested negative upon screening and went on to test positive at the end of quarantine. Most of the subjects had asymptomatic infection (*n* = 145, 78.8%) and were females (*n* = 115, 62.5%). The number of subjects progressively increased across increasing age categories, with 22.3% (*n* = 41) of the subjects being in the ≥60 years category.

We did not find age categories or sex to significantly affect the odds of being symptomatic in the study sample, although people in the category of 50–59 years of age seemed to be more likely to be symptomatic (OR = 1.5, 95% CI = 0.4–5.3) after adjusting for differences in sex (Table 1).

**Table 1:** Subject characteristics and odds of being symptomatic.

	**N (184)**	**%**	**Odds of being symptomatic [OR (95% CI)]**
**Sex**			
Males	69	37.5	1.1 (0.5–2.4)^**a**^
Females	115	62.5	Reference group
**Age group**			
0–29	25	13.6	Reference group
30–39	23	12.5	0.4 (0.1–1.5)^**b**^
40–49	39	21.2	1.2 (0.3–4.3)^**b**^
50–59	56	30.4	1.5 (0.4–5.3)^**b**^
60+	41	22.3	0.8 (0.2–2.6)^**b**^
**Symptoms**			
Asymptomatic	145	78.8	NA
Symptomatic	39	21.2	NA

NA, not applicable.

a Adjusted for age group.

b *Adjusted for sex*.

Median time until viral clearance was similar for males and females (Table 2) and showed an increasing, though nonsignificant, trend across increasing age categories from 11 days in individuals of 0–29 years to 13 days in individuals of ≥60 years. Symptomatic and asymptomatic individuals had the same median time till viral clearance of 11 (*p* = 0.491) days. To adjust for effects of age and sex, we computed hazards of viral clearance among the study subjects (Table 2) and did not see any difference between symptomatic and asymptomatic individuals (hazard ratio for symptomatic individuals compared with asymptomatic ones = 1.08, 85% CI = 0.7–1.5).

**Table 2:** Median time till viral clearance.

	***N* (184)**	**Hazards of viral clearance^**a**^ [HR (95% CI)]**	**Time till viral clearance [median (IQR)]**	***p*-value—log rank test**
**Sex**				
Males	69	1.1 (0.7–1.5)	11 (8–14)	0.622
Females	115	Reference group	12 (10–14)	
**Age group**				
0–29	25	Reference group	11 (8–13)	0.265
30–39	23	0.7 (0.4–1.3)	11 (8–13)	
40–49	39	0.8 (0.5–1.4)	11 (8–13)	
50–59	56	0.7 (0.4–1.2)	12 (9–14.5)	
60+	41	0.6 (0.4–1.0)	13 (9–14)	
**Symptoms**				
Asymptomatic	145	Reference group	11 (8–14)	0.491
Symptomatic	39	1.08 (0.7–1.5)	11 (9–14)	

a *Adjusted for all predictor variables*.

These results indicate that after age and sex were controlled for, time till viral negativity of subclinical infection is similar to that of a non-critically overt one.

## Discussion and Conclusion

To our knowledge, this is the first study reporting time till viral clearance of SARS-CoV-2 RNA in asymptomatic individuals and is the first of its kind analysis to uncover the finding that duration of viral positivity is similar for subclinical and overt infections after controlling for age and sex. Chang et al. (7) have reported the median time till viral in symptomatic patients to be 10.5 days, which is similar to our finding of 11 days in symptomatic individuals and not too different from that of asymptomatic individuals (10 days). Hu et al. (8) have reported the median time till negative convergence in symptomatic patients to be 14 days, which makes sense given the study participants were all highly symptomatic hospitalized patients. The authors also reported age of more than 45 years to be significantly associated with longer duration of viral clearance (HR: 0.37) in these patients. Although the present study saw an increasing trend in time till viral negativity with increasing age categories (HR = 0.8 for age category 40–50 years, HR = 0.7 for 50–60 years, HR = 0.6 for >60 years category), the trend was not statistically significant. This could be partly explained by the difference in severity between participants in our study and those studied by Hu et al., our study sample consisted of non-critical symptomatic and asymptomatic individuals, whereas participants of Hu et al. were symptomatic to the point of being hospitalized. When viewed in conjunction with the finding that asymptomatic and symptomatic individuals have similar viral loads as reported by Zou et al. (9), our results raise questions on similarities in duration of infectiousness between symptomatic and asymptomatic subjects. However, recent reports suggest that viral nucleic acids are still present in patient samples; therefore, asymptomatic patients may shed non-viable virus (especially those with low viral loads/high CT values threshold) that may culture negative though persistently test RT-PCR positive (10). One of the limitations in the present study is that in individuals who were symptomatic upon arrival, it was not possible to ascertain how long they would have been presymptomatic before they landed in Bahrain. This could have affected the estimates of duration of viral positivity in symptomatic patients and warrants further research. Aside from this, these insights shed light on why asymptomatic spread is still very much a substantial risk and underscore the importance of not easing public health preventative measures as yet, such as restrictions on mass gathering, social distancing, and especially opening of businesses that would make population adherence to these measures difficult.

## Data Availability Statement

The raw data supporting the conclusions of this article will be made available by the authors, without undue reservation.

## Ethics Statement

The studies involving human participants were reviewed and approved by Bahrain National Covid-19 Taskforce Ethics Committee. The patients/participants provided their written informed consent to participate in this study.

## Author Contributions

NK: data analysis, interpretation, and preparation of the manuscript. AA and SA: conception and design, data collection, and manuscript review. SO and SLA: writing and manuscript review. MA: conception and design of the study and manuscript review. All authors contributed to the article and approved the submitted version.

## Conflict of Interest

The authors declare that the research was conducted in the absence of any commercial or financial relationships that could be construed as a potential conflict of interest.

## References

[1] Nishiura H Kobayashi T Miyama T Suzuki A Jung SM Hayashi K. Estimation of the asymptomatic ratio of novel coronavirus infections (COVID-19). Int J Infect Dis. (2020) 94:154–5. 10.1016/j.ijid.2020.03.020 32179137 PMC7270890

[2] Levenson E. UNC-Chapel Hill Reverses Plans for in-Person Classes after 130 Students Test Positive for Covid-19. CNN, Cable News Network (2020, August 18). Available online at: edition.cnn.com/2020/08/17/us/coronavirus-college-university/index.html.

[3] Cai W Ivory D Semple K Smith M Lemonides A Higgins L. Tracking Coronavirus Cases at U.S. Colleges and Universities. The New York Times (2020, August 26). Available online at: www.nytimes.com/interactive/2020/us/covid-college-cases-tracker.html.

[4] Kumar S. Eat Out to Help Out' policy failed United Kingdom. The Daily Targum. (2020). Available online at: https://www.dailytargum.com/article/2020/09/kumar-eat-out-to-help-out-policy-failed-united-kingdom. (accessed September 15, 2020).

[5] Arons MM Hatfield KM Reddy SC Kimball A James A Jacobs JR. Presymptomatic SARS-CoV-2 infections and transmission in a skilled nursing facility. N Engl J Med. (2020) 382:2081–90. 10.1056/NEJMoa2008457 32329971 PMC7200056

[6] Mizumoto K Kagaya K Zarebski A Chowell G. Estimating the asymptomatic proportion of coronavirus disease 2019 (COVID-19) cases on board the Diamond Princess cruise ship, Yokohama, Japan, 2020. Euro Surveill. (2020). 25:2000180. 10.2807/1560-7917.ES.2020.25.10.2000180 32183930 PMC7078829

[7] Chang D Mo G Yuan X Tao Y Peng X Wang FS. Time kinetics of viral clearance and resolution of symptoms in novel coronavirus infection. Am J Respir Crit Care Med. (2020) 201:1150–2. 10.1164/rccm.202003-0524LE 32200654 PMC7193851

[8] Hu X Xing Y Jia J Ni W Liang J Zhao D. Factors associated with negative conversion of viral RNA in patients hospitalized with COVID-19. Sci Total Environ. (2020) 728:138812. 10.1016/j.scitotenv.2020.138812 32335406 PMC7175870

[9] Zou L Ruan F Huang M Liang L Huang H Hong Z. SARS-CoV-2 viral load in upper respiratory specimens of infected patients. N Engl J Med. (2020) 382:1177–9. 10.1056/NEJMc2001737 32074444 PMC7121626

[10] Xiao AT Tong YX Zhang S. False negative of RT-PCR and prolonged nucleic acid conversion in COVID-19: rather than recurrence. J Med Virol. (2020) 92:1755–6. 10.1002/jmv.25855 32270882 PMC7262304

